# New-Onset Nonarteritic Anterior Ischemic Optic Neuropathy and Initiators of Semaglutide in US Veterans With Type 2 Diabetes

**DOI:** 10.1001/jamaophthalmol.2025.6262

**Published:** 2026-02-12

**Authors:** Kent Heberer, Adam P. Bress, Steven Cogill, Ana I. Maldonado, Sun H. Kim, Shriram Nallamshetty, Ying Q. Chen, Mei-Chiung Shih, Julie A. Lynch, Jennifer S. Lee

**Affiliations:** 1 VA Palo Alto Healthcare System, Palo Alto, California; 2 VA Palo Alto Cooperative Studies Program Coordinating Center, Palo Alto, California; 3 VA Salt Lake City Healthcare System, Salt Lake City, Utah; 4 Department of Population Health Sciences, School of Medicine, University of Utah, Salt Lake City; 5 Division of Endocrinology, Gerontology, and Metabolism, Department of Medicine, Stanford University School of Medicine, Stanford, California; 6 Division of Cardiovascular Medicine, Stanford School of Medicine, Stanford, California; 7 Stanford Prevention Research Center, Department of Medicine, Stanford University, Stanford, California; 8 Department of Biomedical Data Science, Stanford University School of Medicine, Stanford, California; 9 Division of Epidemiology, School of Medicine, University of Utah, Salt Lake City

## Abstract

**Importance:**

Glucagon-like peptide-1 receptor agonists (GLP-1RAs) are considered safe, effective medications for type 2 diabetes (T2D) and weight loss, used by millions worldwide. While their cardiometabolic benefits are well established, emerging observations suggest a potential association between GLP-1RA use and new-onset nonarteritic anterior ischemic optic neuropathy (NAION).

**Objective:**

To emulate a target trial evaluating the risk of NAION associated with initiation of semaglutide (GLP-1RA), compared with a sodium-glucose cotransporter-2 inhibitor (SGLT2i) as second-line therapy for T2D in a nationwide cohort of US veterans.

**Design, Setting, and Participants:**

This study was conducted nationwide using data from the Veterans Health Administration health care system between March 1, 2018, and March 1, 2025. This active-comparator, new-user, target trial emulation used cause-specific hazard ratios (HRs) that were estimated using overlap weighting to account for confounding. Participants included US veterans with T2D, current metformin use, and no prior GLP-1RA or SGLT2i use. Data analysis was conducted from July 2025 through September 2025.

**Exposure:**

Initiation of semaglutide or any SGLT2i.

**Main Outcome and Measure:**

Incident NAION, identified using *International Statistical Classification of Diseases and Related Health Problems, Tenth Revision* and Systematized Nomenclature of Medicine codes.

**Results:**

A total of 102 361 US veterans met inclusion criteria, including 11 478 initiators of semaglutide and 90 883 initiators of an SGLT2i. Baseline characteristics were well balanced between treatment groups after overlap weighting (mean [SD] age, 60.1 [11.7] years; body mass index, 37.8 [6.7]; hemoglobin A_1c_, 7.0% [1.4]; 85.5% male and 14.5% female; 20.7% Black, 8.1% Hispanic, and 61.9% non-Hispanic White). Over a maximum follow-up of 7.5 years, 173 total incident NAION events occurred. The incidence rate of NAION was 123 per 100 000 person-years among semaglutide initiators and 67 per 100 000 person-years among SGLT2i. In 2.1 years of median follow-up, semaglutide initiators had a 2.33-fold higher risk than SGLT2i initiators (hazard ratio, 2.33; 95% CI, 1.54-3.54; *P* < .001). The overlap weighted incidence rate of NAION was 0.29% for semaglutide initiators and 0.13% for SGLT2i initiators, with a corresponding average treatment effect of 0.16 percentage points.

**Conclusions and Relevance:**

In this nationwide cohort of US veterans with T2D, semaglutide initiators had a 2-fold NAION risk than SGLT2i initiators, while the absolute risk was low. Clinicians and patients should be counseled on the rare but evident increased risk of NAION after semaglutide initiation.

## Key Points

**Question:**

Is semaglutide associated with an increased risk of incidence of nonarteritic anterior ischemic optic neuropathy (NAION) compared with sodium-glucose cotransporter-2 inhibitors (SGLT2i) in patients with type 2 diabetes?

**Findings:**

In this study, among a group of US veterans with type 2 diabetes, initiation of semaglutide was associated with a 2-fold higher risk of incident NAION compared with initiation of an SGLT2i, with overlap weighted cumulative risks of 0.29% vs 0.13% over a median follow-up of 2.1 years.

**Meaning:**

While absolute incidence of NAION is low in patients initiating semaglutide, medical counseling about this potential complication, which can result in substantial loss of vision, may be warranted.

## Teaser

This observational study emulates a target trial evaluating the risk of NAION associated with initiation of semaglutide, compared with a sodium-glucose cotransporter-2 inhibitor as second-line therapy for type 2 diabetes in a nationwide cohort of US veterans.

## Introduction

Semaglutide, a glucagon-like peptide-1 receptor agonist (GLP-1RA), is increasingly used for type 2 diabetes (T2D) and obesity, with 15 million US adults taking it.^1^ Although effective for glycemic control, weight loss, and cardiovascular risk reduction, nonarteritic anterior ischemic optic neuropathy (NAION) has emerged as a serious rare adverse event. Studies show conflicting associations.^2,3,4,5,6,7,8,9^ To address limitations of heterogeneous data sources, design quality, low NAION event rate, and short follow-up, we emulated a target trial within the Veterans Health Administration, US’s largest integrated health care system from 2018 through 2025.

## Methods

### Study Design

This observation study emulating a target trial assessed NAION risk in patients initiating semaglutide vs sodium-glucose cotransporter-2 inhibitors (SGLT2i), using pharmacy dispensing data and detailed clinical covariates, under Strengthening the Reporting of Observational Studies in Epidemiology (STROBE) reporting guidelines and Transparent Reporting of Studies Emulating a Target trial (TARGET) guidelines. We used an active-comparator new-user design with overlap weighting in a retrospective nationwide cohort of US veterans with T2D. Per target trial intention-to-treat protocol, exposure was defined by drug initiation. Risk estimates reflect the initiation effects, not cumulative dose, duration, or time-varying use.

The study was conducted under the Cooperative Studies Program 2012 protocol approved by the US Veterans Affairs Central institutional review board, which waived informed consent due to minimal individual privacy risk (protocol number 17-24). All methodological decisions were finalized a priori, without a stand-alone protocol, to minimize analytic flexibility.

### Participants and Exposure

We included US veterans aged 18 years or older with T2D, taking metformin, who initiated semaglutide or SGLT2i between March 1, 2018, and March 1, 2025 (dual initiators excluded). Index date was the first outpatient pharmacy fill. Eligibility required 1 or more *International Statistical Classification of Diseases and Related Health Problems, Tenth Revision (ICD-10) *codes for T2D within 2 years preindex and metformin fill within 90 days prior. Exclusions were prior type 1 diabetes or secondary diabetes, exposure to other antidiabetic drugs or 30 or more days’ insulin use within 10 years, and prior Systematized Nomenclature of Medicine (SNOMED) or *ICD9/10* codes for NAION, giant cell arteritis, or traumatic optic nerve injury within 10 years.

### Follow-Up and Outcomes

Follow-up spanned from index date until first occurrence of NAION, censoring event (death or competing outcome of giant cell arteritis or traumatic optic nerve injury), or study end (July 1, 2025). Primary outcome was incident NAION, defined as 1 or more clinical encounter with SNOMED code (14357004) or *ICD-10* code (H47.01).

### Baseline Covariates

Baseline covariates were selected a priori for potential to confound the semaglutide-NAION association. Covariates assessed at index date included sociodemographics and treatment initiation year. Clinical cardiometabolic risk indicators and comorbidities in the Charlson Comorbidity Index (by *ICD-9/10* codes) were identified closest to and within 2 years preindex date. Medication use meant 1 or more prescriptions filled within 2 years preindex for phosphodiesterase-5 inhibitors, amiodarone, statins, β-blockers, angiotensin-converting enzyme inhibitors, and angiotensin receptor blockers.

### Statistical Analysis

To address measured baseline confounding, we estimated propensity scores for semaglutide initiation using logistic regression with all baseline covariates,^10^ applying overlap weighting via PSweight R package.^11^ Covariate balance was assessed using absolute standardized mean differences, with less than 0.01 considered adequate. We calculated cumulative incidence (events/persons at-risk) and incidence rates (events/person-time) of NAION by treatment group. Kaplan-Meier estimates were compared using log-rank test for unweighted and overlap-weighted cohorts. Under intention to treat, adjusted hazard ratios (HRs) and 95% CIs were estimated using Cox proportional hazards models incorporating propensity score-based overlap weighting, including the baseline covariates. Baseline covariates with less than 20% missingness (judged missing at random) were imputed using multiple imputation with chained equations via mice R Package,^12^ generating 10 imputed datasets.

## Results

### Study Population

**Figure 1.**
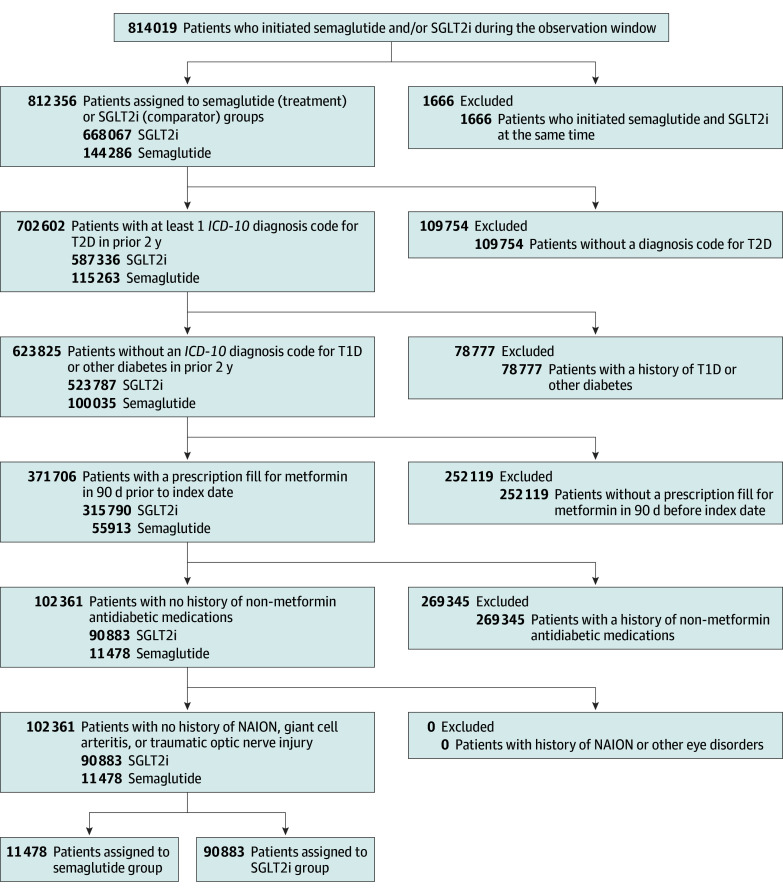
Patient Cohort Selection Flow Diagram Depiction of the cohort selection logic with inclusion and exclusion criteria, starting with 814 019 patients who initiated a semaglutide or sodium-glucose cotransport protein 2 inhibitors (SGLT2i) treatment regimen during the enrollment window and ending with 102 361 patients in the analytic cohort. *ICD-10 *indicates* International Statistical Classification of Diseases and Related Health Problems, Tenth Revision*; NAION, nonarteritic ischemic optic neuropathy; T1D, type 1 diabetes; T2D, type 2 diabetes.

Among 814 019 veterans who initiated semaglutide or an SGLT2i between March 1, 2018, and March 1, 2025, 102 361 met study eligibility criteria (Figure 1). Of these, 11 478 initiated semaglutide and 90 883 initiated SGLT2i. Almost all SGLT2i initiators received empagliflozin (n = 90 816). The remainder (n = 67) received dapagliflozin or canagliflozin.

### Baseline Characteristics

At treatment initiation, semaglutide users were younger (mean [SD] age, 59.2 [11.7] vs 64.6 [11.9] years), had higher body mass index (calculated as weight in kilograms divided by height in meters squared) (38.8 [7.4] vs 33.6 [6.4]), and had lower hemoglobin A_1c_ levels (7.0% [1.4] vs 7.3% [1.5]) than SGLT2i users. They were more likely female (17.2% vs 6.3%) and Black (21.6% vs 17.8%). After overlap weighting, all covariates were balanced, with standardized mean differences less than 0.01 (Table).

**Table.** Baseline Covariates Between Initiators of Semaglutide and a Sodium-Glucose Cotransporter Inhibitor (SGLT2i) Before and After Overlap Weighting

Characteristics^a^	Before weighting, No. (%)		SMD	After weighting,^b^ %		SMD^c^
	Semaglutide (n = 11 478)	SGLT2i (n = 90 883)		Semaglutide	SGLT2i	
Age, y, mean (SD)	59.2 (11.7)	64.6 (11.9)	0.46	60.1 (11.7)	60.1 (12.2)	1.0 × 10^−12^
Sex						
Female	1978 (17.2)	5749 (6.3)	0.34	14.5	14.5	1.0 × 10^−12^
Male	9500 (82.8)	85 134 (93.7)	NA	85.5	85.5	NA
Race and ethnicity (reference: non-Hispanic White)						
American Indian or Alaska Native	66 (0.6)	641 (0.7)	0.02	0.6	0.6	1.5 × 10^−12^
Asian	132 (1.2)	1600 (1.8)	0.05	1.2	1.2	1.7 × 10^−12^
Black or African American	2480 (21.6)	16 218 (17.8)	0.09	20.7	20.7	1.5 × 10^−12^
Hispanic or Latino	948 (8.3)	6951 (7.6)	0.02	8.1	8.1	2.5 × 10^−12^
Hawaiian or Pacific Islander	131 (1.1)	979 (1.1)	0.01	1.1	1.1	3.3 × 10^−13^
Non-Hispanic White	6992 (60.9)	58 818 (64.7)	NA	61.9	61.9	NA
Other or not reported	729 (6.4)	5676 (6.2)	0.00	6.4	6.4	4.2 × 10^−13^
Index year (reference: 2024)						
2018	21 (0.2)	887 (1.0)	0.10	0.2	0.2	3.4 × 10^−10^
2019	192 (1.7)	2592 (2.9)	0.08	1.9	1.9	2.2 × 10^−12^
2020	321 (2.8)	4677 (5.1)	0.12	3.1	3.1	2.9 × 10^−12^
2021	1190 (10.4)	10 746 (11.8)	0.05	10.8	10.8	5.5 × 10^−12^
2022	1960 (17.1)	17 915 (19.7)	0.07	17.5	17.5	7.3 × 10^−12^
2023	3177 (27.7)	23 864 (26.3)	0.03	27.5	27.5	9.8 × 10^−12^
2024	4167 (36.3)	26 139 (28.8)	NA	35.0	35.0	NA
2025	450 (3.9)	4063 (4.5)	0.03	4.0	4.0	3.3 × 10^−12^
Clinical and laboratory measurements, mean (SD)						
Body mass index,^d^	38.8 (7.4)	33.6 (6.4)	0.74	37.8 (6.7)	37.8 (7.8)	4.4 × 10^−14^
Systolic blood pressure, mm Hg	133.7 (15.8)	135.1 (17.0)	0.09	133.9 (15.9)	133.9 (16.0)	2.1 × 10^−12^
Total cholesterol, mg/dL	167.7 (47.0)	164.0 (48.1)	0.08	166.9 (47.7)	166.9 (45.8)	9.1 × 10^−13^
Triglyceride, mg/dL	188.5 (164.1)	195.8 (175.5)	0.04	191.7 (170.8)	191.7 (151.8)	1.7 × 10^−12^
High-density lipoprotein, mg/dL	41.8 (11.4)	41.3 (11.3)	0.04	41.5 (11.2)	41.5 (11.5)	2.2 × 10^−12^
Low-density lipoprotein, mg/dL	94.1 (38.6)	89.9 (38.9)	0.11	93.2 (38.9)	93.2 (38.3)	1.6 × 10^−12^
Hemoglobin A_1c_, %	7.0 (1.4)	7.3 (1.5)	0.23	7.0 (1.4)	7.0 (1.2)	5.9 × 10^−13^
Lifestyle factors						
AUDIT-C^e^	1.5 (2.1)	1.7 (2.5)	0.08	1.5 (2.2)	1.5 (2.3)	1.2 × 10^−12^
Smoking history, yes	5797 (50.5)	49 827 (54.8)	0.09	51.0	51.0	3.1 × 10^−13^
Charlson Comorbidity Index						
Cerebrovascular disease	618 (5.4)	6702 (7.4)	0.08	5.7	5.7	1.2 × 10^−13^
Congestive heart failure	807 (7.0)	12 882 (14.2)	0.23	7.7	7.7	3.9 × 10^−13^
Chronic pulmonary disease	2219 (19.3)	17 010 (18.7)	0.02	19.0	19.0	8.3 × 10^−13^
Dementia	210 (1.8)	2306 (2.5)	0.05	1.9	1.9	4.6 × 10^−14^
Diabetes with complications	3591 (31.3)	32 079 (35.3)	0.09	32.2	32.2	2.5 × 10^−12^
Hemiplegia or paraplegia	90 (0.8)	435 (0.5)	0.04	0.7	0.7	4.9 × 10^−13^
HIV or AIDS	87 (0.8)	377 (0.4)	0.04	0.7	0.7	7.5 × 10^−14^
Liver disease (mild)	1802 (15.7)	8954 (9.9)	0.18	14.6	14.6	2.8 × 10^−12^
Liver disease (severe)	146 (1.3)	1192 (1.3)	0.00	1.3	1.3	1.9 × 10^−13^
Malignancy	1576 (13.7)	13 724 (15.1)	0.04	13.9	13.9	1.3 × 10^−12^
Metastasis	67 (0.6)	557 (0.6)	0.00	0.6	0.6	7.6 × 10^−13^
Myocardial infarction	305 (2.7)	4221 (4.6)	0.11	2.9	2.9	1.1 × 10^−12^
Peptic ulcer	50 (0.4)	591 (0.7)	0.03	0.5	0.5	8.2 × 10^−13^
Peripheral vascular disease	713 (6.2)	7810 (8.6)	0.09	6.5	6.5	1.6 × 10^−13^
Kidney disease	880 (7.7)	9004 (9.9)	0.08	8.0	8.0	2.1 × 10^−12^
Rheumatic disorders	209 (1.8)	1325 (1.5)	0.03	1.7	1.7	1.3 × 10^−13^

Abbreviations: AUDIT-C, Alcohol Use Disorders Identification Test—Consumption; NA, not applicable; SMD, standardized mean difference.

^a^ Values were imputed for the following (semaglutide/SGLT2i): body mass index = 454 of 4222, total cholesterol = 952 of 6989, triglycerides = 1047 of 7945, high-density lipoprotein = 962 of 7099, low-density lipoprotein = 1048 of 7863, blood pressure = 387 of 3122, hemoglobin A_1c_ = 307 of 2373, AUDIT-C = 137 of 1387.

^b^ Data for before weighting are presented as number (percentage) of participants and data for after weighting as percentage of participants unless otherwise indicated. The total numbers of patients in the postoverlap weighted columns were omitted because the numbers were slightly different as a result of the weighting.

^c^ All values less than .01.

^d^ Calculated as weight in kilograms divided by height in meters squared.

^e^ AUDIT-C raw scores range from 0-12, with 0 indicating no drinking behavior.

### Primary Outcome

**Figure 2.**
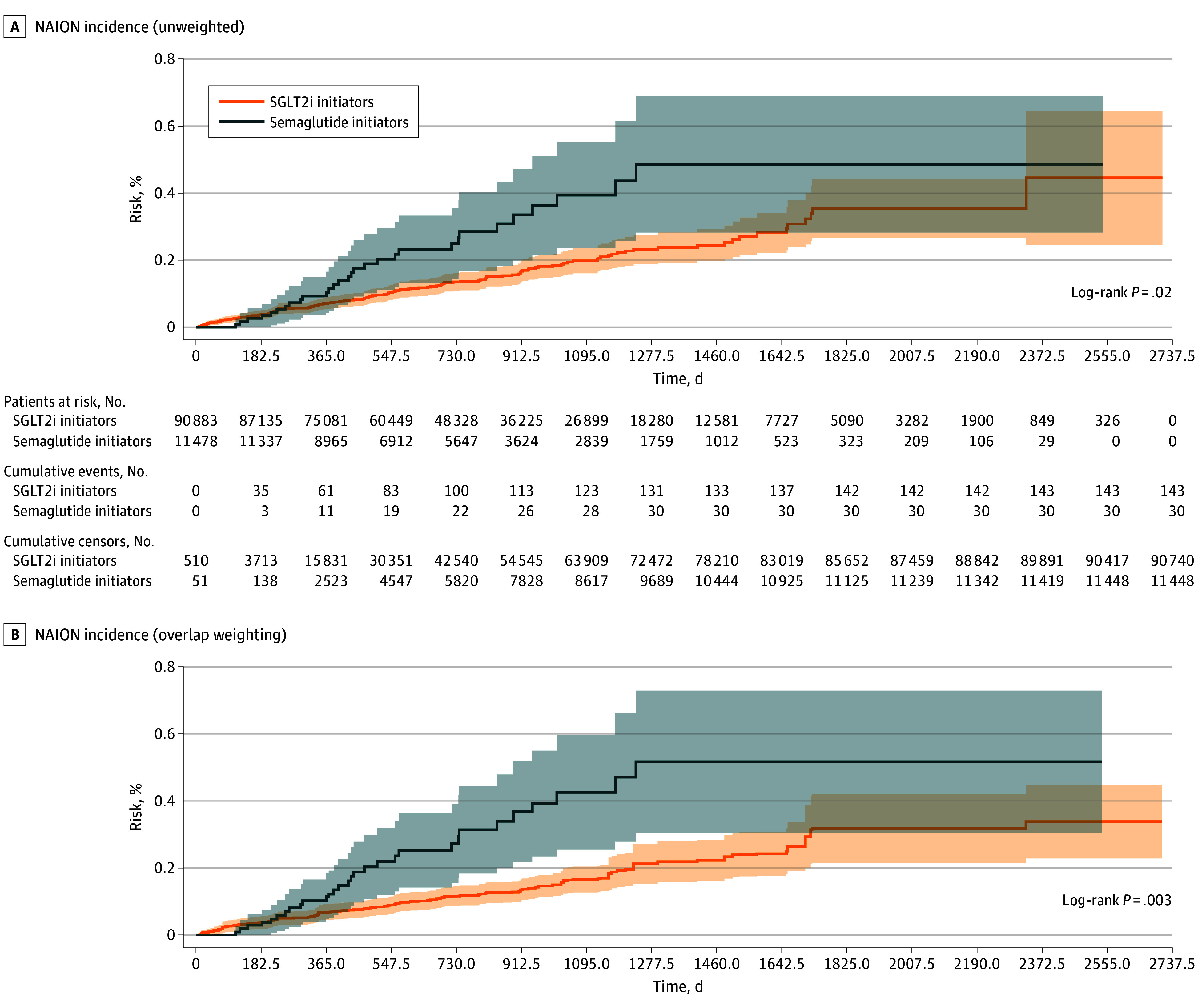
Risk Curves of New-Onset Nonarteritic Ischemic Optic Neuropathy (NAION) in the Unweighted Cohort and the Overlap Weighted Cohort The middle portion shows the number of patients at risk, number of NAION events, and number of censored events at 6-month intervals for the unweighted cohort. The incidence of NAION is significantly higher in the semaglutide group compared with the sodium-glucose cotransport protein 2 inhibitors (SGLT2i) group before and after weighting. With overlap weighting, compared with without overlap weighting, the estimated risk of new-onset NAION is elevated in the semaglutide initiators group and reduced in the SLGT2i initiators group. Significance in risk between the 2 groups was assessed using robust estimates from the log-rank test.

Over 2.1 years of median follow-up spanning 239 333 person-years (24 416 for semaglutide, 214 917 for SGLT2i), 30 semaglutide initiators developed NAION (incidence rate, 123 per 100 000 person-years) vs 143 SGLT2i initiators (67 per 100 000 person-years). Kaplan-Meier curves demonstrated early and persistent separation, with cumulative incidence consistently higher among semaglutide initiators (Figure 2). In unweighted analyses, semaglutide initiation was associated with increased NAION hazard than SGLT2i initiation (HR, 1.84; 95% CI, 1.24-2.73; *P* = .002). After overlap weighting, semaglutide initiators had 2.33-fold higher NAION hazard (HR, 2.33; 95% CI, 1.53-3.54; *P* < .001). Overlap-weighted NAION incidence over a maximum 7.5 years’ follow-up was 0.29% for semaglutide initiators vs 0.13% for SGLT2i initiators.

## Discussion

During 2018 through 2025, semaglutide initiators among US veterans with T2D taking metformin had a 2-fold higher NAION risk than SGLT2i in 2.1 years of median follow-up after extensive covariate adjustment (overlap-weighted cumulative absolute risks: 0.3% vs 0.1%).

Findings align with growing evidence linking GLP-1RAs, particularly semaglutide, to NAION. Large observational analyses reported similar 2- to 3-fold risks,^2,3,4,5^ whereas others observed attenuated or null associations.^6,7,8,9^ Absolute incidence remains low (approximately 1 additional case per several thousand treated patients).^13^^.^

Prior inconsistences in electronic health record studies include NAION definitions, comparator choice, new-user designs, follow-up length, sampling approaches, confounder adjustments, and NAION rarity. We leveraged US’s largest integrated health system, with extended follow-up to 7 years, extensive confounder adjustment, and relatively large number of incident NAION events. Outpatient pharmacy dispensing, not prescription orders or claims, defined medication use. We incorporated detailed clinical covariates, including cardiometabolic risk measures and comorbidities. We nearly doubled the semaglutide cohort and accrued 6-fold more person-years (24 416) than the prior Observational Health Data Sciences and Informatics pooled analysis (6824 semaglutide initiators [4429 person-years] and 61 916 empagliflozin initiators [46 496 person-years]).^8^

As clinicians inform patients on semaglutide’s meaningful cardiometabolic benefits, they should also counsel on NAION as a rare, serious vision-loss event. Consistent with recent commentaries,^2,3,4,5,6,7,8,9,13,14,15^ clinicians should encourage prompt evaluation of visual symptoms and consider ocular risk factors (eg, optic nerve disease, prior NAION). Ophthalmologists should identify semaglutide use in patients with NAION, who should inform their prescribing clinicians.^14^

### Limitations

Limitations include potential residual unmeasured confounding; for example, from optic nerve head anatomy, cup-to-disc ratio, NAION family history, sleep apnea severity, nocturnal hypotension, diabetes duration, non-VA care. Future per-protocol effect studies should characterize dose or duration response relationships. Generalizability may be limited due to older age, predominance of males, and higher cardiometabolic risk among veterans than the general US population. While NAION’s positive predictive value from 1 or more *ICD-10* coding is 74.5% using medical records,^16^ outcome misclassification could occur from miscoding, misdiagnosis, or missingness. This could affect overall incidence rates but likely nondifferentially bias the HR toward the null. The biological mechanism linking GLP-1RAs to NAION remains unclear, including hypotension, volume depletion from gastrointestinal adverse effects, rapid glycemic improvement with transient microvascular dysregulation, and impaired vascular autoregulation at the optic nerve head.^15,17^

## Conclusions

In conclusion, veterans with T2D taking metformin who initiated semaglutide had a 2-fold higher NAION risk than those who initiated SGLT2i, although absolute risk was low. This study supports medical counseling about NAION risk and implications for vision loss after semaglutide initiation.

## Notes

Supplement. Data sharing statement
