# Supplementary material for: New-Onset Nonarteritic Anterior Ischemic Optic Neuropathy and Initiators of Semaglutide in US Veterans With Type 2 Diabetes
Source: JAMA Ophthalmol. 2026 Feb 12;144(3):259–64. doi: 10.1001/jamaophthalmol.2025.6262 (PMC12902922; doi:10.1001/jamaophthalmol.2025.6262)
Supplement: Supplement. — Data sharing statement [file jamaophthalmol-e256262-s001.pdf]

Data Sharing Statement

Heberer. New Onset Nonarteritic Anterior Ischemic Optic Neuropathy and Initiators of Semaglutide in US Veterans With Type 2 Diabetes. *JAMA Ophthalmol*. Published February 12, 2026. doi:10.1001/jamaophthalmol.2025.6262

Data
Data available: Yes
Data types: Deidentified participant data, Data (not involving human participants), Data dictionary
How to access data: Patient-level data are already accessible to all VA researchers with appropriate IRB approvals.
When available: With publication

Supporting Documents
Document types: None

Additional Information
Who can access the data: Patient-level data are already accessible to all VA researchers with appropriate IRB approvals.
Types of analyses: Data are available for any type of analysis.
Mechanisms of data availability: Data will be made available after IRB approval.
